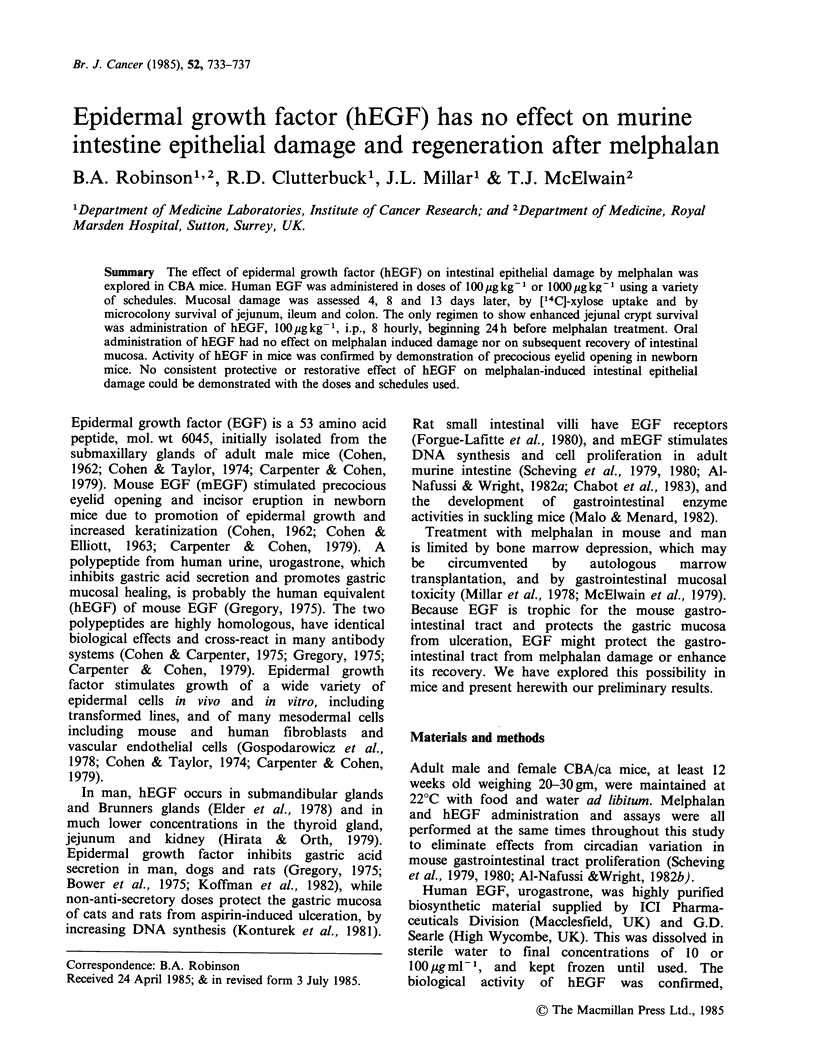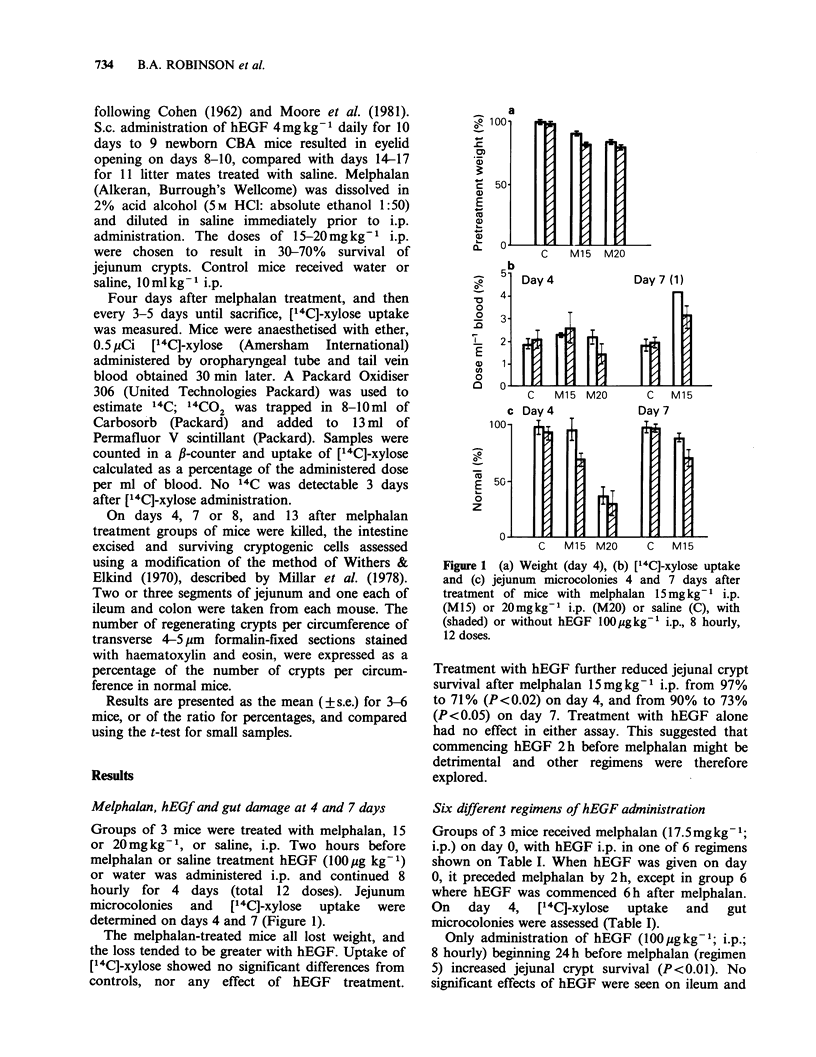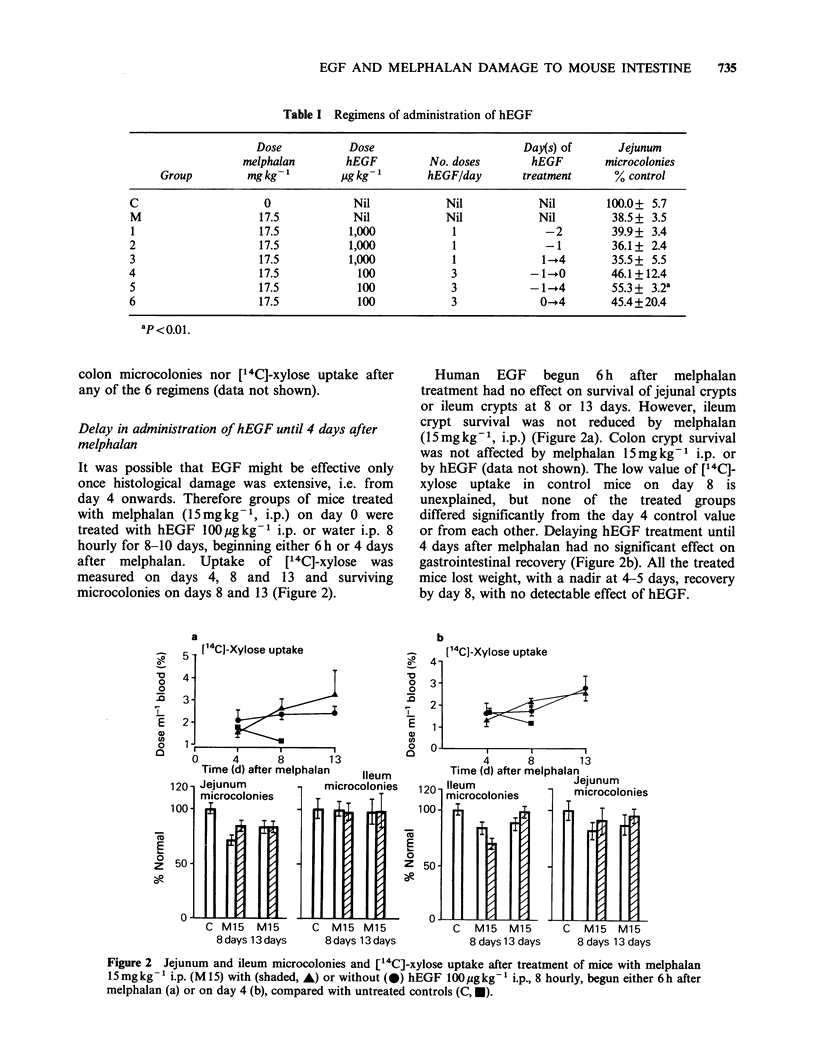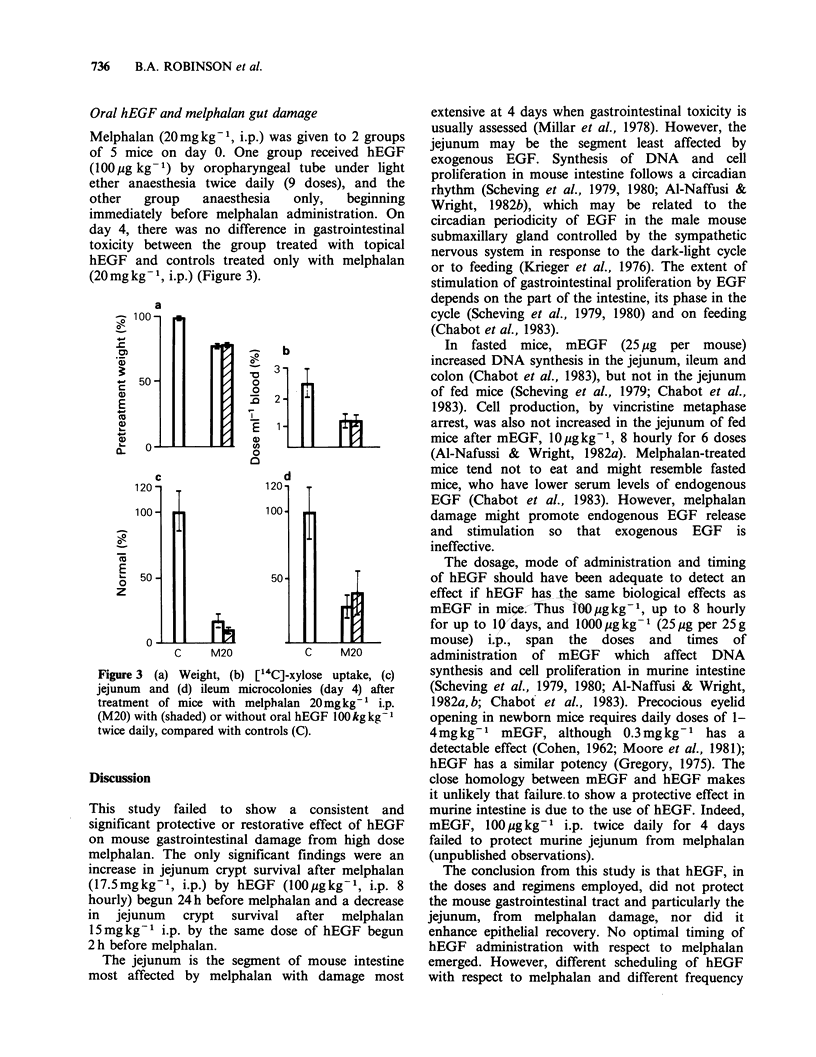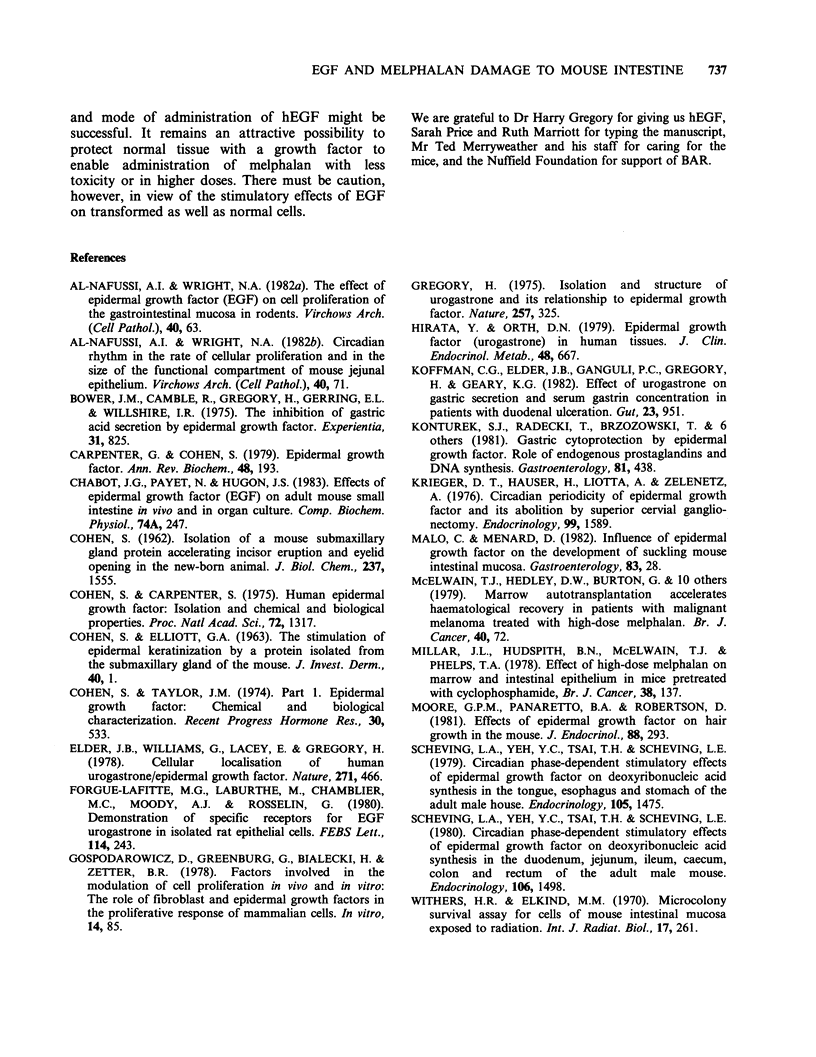# Epidermal growth factor (hEGF) has no effect on murine intestine epithelial damage and regeneration after melphalan.

**DOI:** 10.1038/bjc.1985.251

**Published:** 1985-11

**Authors:** B. A. Robinson, R. D. Clutterbuck, J. L. Millar, T. J. McElwain

## Abstract

The effect of epidermal growth factor (hEGF) on intestinal epithelial damage by melphalan was explored in CBA mice. Human EGF was administered in doses of 100 micrograms kg-1 or 1000 micrograms kg-1 using a variety of schedules. Mucosal damage was assessed 4, 8 and 13 days later, by [14C]-xylose uptake and by microcolony survival of jejunum, ileum and colon. The only regimen to show enhanced jejunal crypt survival was administration of hEGF, 100 micrograms kg-1, i.p., 8 hourly, beginning 24 h before melphalan treatment. Oral administration of hEGF had no effect on melphalan induced damage nor on subsequent recovery of intestinal mucosa. Activity of hEGF in mice was confirmed by demonstration of precocious eyelid opening in newborn mice. No consistent protective or restorative effect of hEGF on melphalan-induced intestinal epithelial damage could be demonstrated with the doses and schedules used.


*Br. J. Cancer* (1985), **52**, 733–737

### Epidermal growth factor (hEGF) has no effect on murine intestine epithelial damage and regeneration after melphalan

B.A. Robinson[1,2], R.D. Clutterbuck[1], J.L. Millar[1] & T.J. McElwain[2]

[1]*Department of Medicine Laboratories, Institute of Cancer Research; and* [2]*Department of Medicine, Royal Marsden Hospital, Sutton, Surrey, UK.*

**Summary** The effect of epidermal growth factor (hEGF) on intestinal epithelial damage by melphalan was explored in CBA mice. Human EGF was administered in doses of $100 \mu g kg^{-1}$ or $1000 \mu g kg^{-1}$ using a variety of schedules. Mucosal damage was assessed 4, 8 and 13 days later, by [$^{14}$C]-xylose uptake and by microcolony survival of jejunum, ileum and colon. The only regimen to show enhanced jejunal crypt survival was administration of hEGF, $100 \mu g kg^{-1}$, i.p., 8 hourly, beginning 24 h before melphalan treatment. Oral administration of hEGF had no effect on melphalan induced damage nor on subsequent recovery of intestinal mucosa. Activity of hEGF in mice was confirmed by demonstration of precocious eyelid opening in newborn mice. No consistent protective or restorative effect of hEGF on melphalan-induced intestinal epithelial damage could be demonstrated with the doses and schedules used.

Epidermal growth factor (EGF) is a 53 amino acid peptide, mol. wt 6045, initially isolated from the submaxillary glands of adult male mice (Cohen, 1962; Cohen & Taylor, 1974; Carpenter & Cohen, 1979). Mouse EGF (mEGF) stimulated precocious eyelid opening and incisor eruption in newborn mice due to promotion of epidermal growth and increased keratinization (Cohen, 1962; Cohen & Elliott, 1963; Carpenter & Cohen, 1979). A polypeptide from human urine, urogastrone, which inhibits gastric acid secretion and promotes gastric mucosal healing, is probably the human equivalent (hEGF) of mouse EGF (Gregory, 1975). The two polypeptides are highly homologous, have identical biological effects and cross-react in many antibody systems (Cohen & Carpenter, 1975; Gregory, 1975; Carpenter & Cohen, 1979). Epidermal growth factor stimulates growth of a wide variety of epidermal cells *in vivo* and *in vitro,* including transformed lines, and of many mesodermal cells including mouse and human fibroblasts and vascular endothelial cells (Gospodarowicz *et al.,* 1978; Cohen & Taylor, 1974; Carpenter & Cohen, 1979).

In man, hEGF occurs in submandibular glands and Brunners glands (Elder *et al.,* 1978) and in much lower concentrations in the thyroid gland, jejunum and kidney (Hirata & Orth, 1979). Epidermal growth factor inhibits gastric acid secretion in man, dogs and rats (Gregory, 1975; Bower *et al.,* 1975; Koffman *et al.,* 1982), while non-anti-secretory doses protect the gastric mucosa of cats and rats from aspirin-induced ulceration, by increasing DNA synthesis (Konturek *et al.,* 1981). Rat small intestinal villi have EGF receptors (Forgue-Lafitte *et al.,* 1980), and mEGF stimulates DNA synthesis and cell proliferation in adult murine intestine (Scheving *et al.,* 1979, 1980; Al-Nafussi & Wright, 1982*a*; Chabot *et al.,* 1983), and the development of gastrointestinal enzyme activities in suckling mice (Malo & Menard, 1982).

Treatment with melphalan in mouse and man is limited by bone marrow depression, which may be circumvented by autologous marrow transplantation, and by gastrointestinal mucosal toxicity (Millar *et al.,* 1978; McElwain *et al.,* 1979). Because EGF is trophic for the mouse gastro-intestinal tract and protects the gastric mucosa from ulceration, EGF might protect the gastro-intestinal tract from melphalan damage or enhance its recovery. We have explored this possibility in mice and present herewith our preliminary results.

### Materials and methods

Adult male and female CBA/ca mice, at least 12 weeks old weighing 20–30 gm, were maintained at 22°C with food and water *ad libitum.* Melphalan and hEGF administration and assays were all performed at the same times throughout this study to eliminate effects from circadian variation in mouse gastrointestinal tract proliferation (Scheving *et al.,* 1979, 1980; Al-Nafussi &Wright, 1982*b).*

Human EGF, urogastrone, was highly purified biosynthetic material supplied by ICI Pharmaceuticals Division (Macclesfield, UK) and G.D. Searle (High Wycombe, UK). This was dissolved in sterile water to final concentrations of 10 or $100 \mu g ml^{-1}$, and kept frozen until used. The biological activity of hEGF was confirmed,

Correspondence: B.A. Robinson
Received 24 April 1985; & in revised form 3 July 1985.

© The Macmillan Press Ltd., 1985

following Cohen (1962) and Moore et al. (1981). S.c. administration of hEGF 4 mg kg$^{-1}$ daily for 10 days to 9 newborn CBA mice resulted in eyelid opening on days 8–10, compared with days 14–17 for 11 litter mates treated with saline. Melphalan (Alkeran, Burrough's Wellcome) was dissolved in 2% acid alcohol (5 M HCl: absolute ethanol 1:50) and diluted in saline immediately prior to i.p. administration. The doses of 15–20 mg kg$^{-1}$ i.p. were chosen to result in 30–70% survival of jejunum crypts. Control mice received water or saline, 10 ml kg$^{-1}$ i.p.

Four days after melphalan treatment, and then every 3–5 days until sacrifice, [$^{14}$C]-xylose uptake was measured. Mice were anaesthetised with ether, 0.5 $\mu$Ci [$^{14}$C]-xylose (Amersham International) administered by oropharyngeal tube and tail vein blood obtained 30 min later. A Packard Oxidiser 306 (United Technologies Packard) was used to estimate $^{14}$C; $^{14}CO_2$ was trapped in 8–10 ml of Carbosorb (Packard) and added to 13 ml of Permafluor V scintillant (Packard). Samples were counted in a $\beta$-counter and uptake of [$^{14}$C]-xylose calculated as a percentage of the administered dose per ml of blood. No $^{14}$C was detectable 3 days after [$^{14}$C]-xylose administration.

On days 4, 7 or 8, and 13 after melphalan treatment groups of mice were killed, the intestine excised and surviving cryptogenic cells assessed using a modification of the method of Withers & Elkind (1970), described by Millar et al. (1978). Two or three segments of jejunum and one each of ileum and colon were taken from each mouse. The number of regenerating crypts per circumference of transverse 4–5 $\mu$m formalin-fixed sections stained with haematoxylin and eosin, were expressed as a percentage of the number of crypts per circumference in normal mice.

Results are presented as the mean (±s.e.) for 3–6 mice, or of the ratio for percentages, and compared using the $t$-test for small samples.

### Results

### Melphalan, hEGf and gut damage at 4 and 7 days

Groups of 3 mice were treated with melphalan, 15 or 20 mg kg$^{-1}$, or saline, i.p. Two hours before melphalan or saline treatment hEGF (100 $\mu$g kg$^{-1}$) or water was administered i.p. and continued 8 hourly for 4 days (total 12 doses). Jejunum microcolonies and [$^{14}$C]-xylose uptake were determined on days 4 and 7 (Figure 1).

The melphalan-treated mice all lost weight, and the loss tended to be greater with hEGF. Uptake of [$^{14}$C]-xylose showed no significant differences from controls, nor any effect of hEGF treatment.

**Figure 1** (a) Weight (day 4), (b) [$^{14}$C]-xylose uptake and (c) jejunum microcolonies 4 and 7 days after treatment of mice with melphalan 15 mg kg$^{-1}$ i.p. (M15) or 20 mg kg$^{-1}$ i.p. (M20) or saline (C), with (shaded) or without hEGF 100 $\mu$g kg$^{-1}$ i.p., 8 hourly, 12 doses.

Treatment with hEGF further reduced jejunal crypt survival after melphalan 15 mg kg$^{-1}$ i.p. from 97% to 71% ($P<0.02$) on day 4, and from 90% to 73% ($P<0.05$) on day 7. Treatment with hEGF alone had no effect in either assay. This suggested that commencing hEGF 2 h before melphalan might be detrimental and other regimens were therefore explored.

### Six different regimens of hEGF administration

Groups of 3 mice received melphalan (17.5 mg kg$^{-1}$; i.p.) on day 0, with hEGF i.p. in one of 6 regimens shown on Table I. When hEGF was given on day 0, it preceded melphalan by 2 h, except in group 6 where hEGF was commenced 6 h after melphalan. On day 4, [$^{14}$C]-xylose uptake and gut microcolonies were assessed (Table I).

Only administration of hEGF (100 $\mu$g kg$^{-1}$; i.p.; 8 hourly) beginning 24 h before melphalan (regimen 5) increased jejunal crypt survival ($P<0.01$). No significant effects of hEGF were seen on ileum and

**Table I** Regimens of administration of hEGF

| Group | *Dose melphalan mg kg⁻¹* | *Dose hEGF µg kg⁻¹* | *No. doses hEGF/day* | *Day(s) of hEGF treatment* | *Jejunum microcolonies % control* |
|---|---|---|---|---|---|
| C | 0 | Nil | Nil | Nil | 100.0 ± 5.7 |
| M | 17.5 | Nil | Nil | Nil | 38.5 ± 3.5 |
| 1 | 17.5 | 1,000 | 1 | −2 | 39.9 ± 3.4 |
| 2 | 17.5 | 1,000 | 1 | −1 | 36.1 ± 2.4 |
| 3 | 17.5 | 1,000 | 1 | 1→4 | 35.5 ± 5.5 |
| 4 | 17.5 | 100 | 3 | −1→0 | 46.1 ± 12.4 |
| 5 | 17.5 | 100 | 3 | −1→4 | 55.3 ± 3.2[a] |
| 6 | 17.5 | 100 | 3 | 0→4 | 45.4 ± 20.4 |

[a] $P<0.01$.

colon microcolonies nor [¹⁴C]-xylose uptake after any of the 6 regimens (data not shown).

*Delay in administration of hEGF until 4 days after melphalan*

It was possible that EGF might be effective only once histological damage was extensive, i.e. from day 4 onwards. Therefore groups of mice treated with melphalan (15 mg kg$^{-1}$, i.p.) on day 0 were treated with hEGF 100 µg kg$^{-1}$ i.p. or water i.p. 8 hourly for 8–10 days, beginning either 6 h or 4 days after melphalan. Uptake of [¹⁴C]-xylose was measured on days 4, 8 and 13 and surviving microcolonies on days 8 and 13 (Figure 2).

Human EGF begun 6 h after melphalan treatment had no effect on survival of jejunal crypts or ileum crypts at 8 or 13 days. However, ileum crypt survival was not reduced by melphalan (15 mg kg$^{-1}$, i.p.) (Figure 2a). Colon crypt survival was not affected by melphalan 15 mg kg$^{-1}$ i.p. or by hEGF (data not shown). The low value of [¹⁴C]-xylose uptake in control mice on day 8 is unexplained, but none of the treated groups differed significantly from the day 4 control value or from each other. Delaying hEGF treatment until 4 days after melphalan had no significant effect on gastrointestinal recovery (Figure 2b). All the treated mice lost weight, with a nadir at 4–5 days, recovery by day 8, with no detectable effect of hEGF.

**Figure 2** Jejunum and ileum microcolonies and [¹⁴C]-xylose uptake after treatment of mice with melphalan 15 mg kg$^{-1}$ i.p. (M15) with (shaded, ▲) or without (●) hEGF 100 µg kg$^{-1}$ i.p., 8 hourly, begun either 6 h after melphalan (a) or on day 4 (b), compared with untreated controls (C, ■).

*Oral hEGF and melphalan gut damage*

Melphalan (20 mg kg$^{-1}$, i.p.) was given to 2 groups of 5 mice on day 0. One group received hEGF (100 $\mu$g kg$^{-1}$) by oropharyngeal tube under light ether anaesthesia twice daily (9 doses), and the other group anaesthesia only, beginning immediately before melphalan administration. On day 4, there was no difference in gastrointestinal toxicity between the group treated with topical hEGF and controls treated only with melphalan (20 mg kg$^{-1}$, i.p.) (Figure 3).

**Figure 3** (a) Weight, (b) [$^{14}$C]-xylose uptake, (c) jejunum and (d) ileum microcolonies (day 4) after treatment of mice with melphalan 20 mg kg$^{-1}$ i.p. (M20) with (shaded) or without oral hEGF 100 *k*g kg$^{-1}$ twice daily, compared with controls (C).

### Discussion

This study failed to show a consistent and significant protective or restorative effect of hEGF on mouse gastrointestinal damage from high dose melphalan. The only significant findings were an increase in jejunum crypt survival after melphalan (17.5 mg kg$^{-1}$, i.p.) by hEGF (100 $\mu$g kg$^{-1}$, i.p. 8 hourly) begun 24 h before melphalan and a decrease in jejunum crypt survival after melphalan 15 mg kg$^{-1}$ i.p. by the same dose of hEGF begun 2 h before melphalan.

The jejunum is the segment of mouse intestine most affected by melphalan with damage most extensive at 4 days when gastrointestinal toxicity is usually assessed (Millar *et al.*, 1978). However, the jejunum may be the segment least affected by exogenous EGF. Synthesis of DNA and cell proliferation in mouse intestine follows a circadian rhythm (Scheving *et al.*, 1979, 1980; Al-Naffusi & Wright, 1982*b*), which may be related to the circadian periodicity of EGF in the male mouse submaxillary gland controlled by the sympathetic nervous system in response to the dark-light cycle or to feeding (Krieger *et al.*, 1976). The extent of stimulation of gastrointestinal proliferation by EGF depends on the part of the intestine, its phase in the cycle (Scheving *et al.*, 1979, 1980) and on feeding (Chabot *et al.*, 1983).

In fasted mice, mEGF (25 $\mu$g per mouse) increased DNA synthesis in the jejunum, ileum and colon (Chabot *et al.*, 1983), but not in the jejunum of fed mice (Scheving *et al.*, 1979; Chabot *et al.*, 1983). Cell production, by vincristine metaphase arrest, was also not increased in the jejunum of fed mice after mEGF, 10 $\mu$g kg$^{-1}$, 8 hourly for 6 doses (Al-Nafussi & Wright, 1982*a*). Melphalan-treated mice tend not to eat and might resemble fasted mice, who have lower serum levels of endogenous EGF (Chabot *et al.*, 1983). However, melphalan damage might promote endogenous EGF release and stimulation so that exogenous EGF is ineffective.

The dosage, mode of administration and timing of hEGF should have been adequate to detect an effect if hEGF has the same biological effects as mEGF in mice. Thus 100 $\mu$g kg$^{-1}$, up to 8 hourly for up to 10 days, and 1000 $\mu$g kg$^{-1}$ (25 $\mu$g per 25 g mouse) i.p., span the doses and times of administration of mEGF which affect DNA synthesis and cell proliferation in murine intestine (Scheving *et al.*, 1979, 1980; Al-Naffusi & Wright, 1982*a,b*; Chabot *et al.*, 1983). Precocious eyelid opening in newborn mice requires daily doses of 1–4 mg kg$^{-1}$ mEGF, although 0.3 mg kg$^{-1}$ has a detectable effect (Cohen, 1962; Moore *et al.*, 1981); hEGF has a similar potency (Gregory, 1975). The close homology between mEGF and hEGF makes it unlikely that failure to show a protective effect in murine intestine is due to the use of hEGF. Indeed, mEGF, 100 $\mu$g kg$^{-1}$ i.p. twice daily for 4 days failed to protect murine jejunum from melphalan (unpublished observations).

The conclusion from this study is that hEGF, in the doses and regimens employed, did not protect the mouse gastrointestinal tract and particularly the jejunum, from melphalan damage, nor did it enhance epithelial recovery. No optimal timing of hEGF administration with respect to melphalan emerged. However, different scheduling of hEGF with respect to melphalan and different frequency and mode of administration of hEGF might be successful. It remains an attractive possibility to protect normal tissue with a growth factor to enable administration of melphalan with less toxicity or in higher doses. There must be caution, however, in view of the stimulatory effects of EGF on transformed as well as normal cells.

We are grateful to Dr Harry Gregory for giving us hEGF, Sarah Price and Ruth Marriott for typing the manuscript, Mr Ted Merryweather and his staff for caring for the mice, and the Nuffield Foundation for support of BAR.

**References**

AL-NAFUSSI, A.I. & WRIGHT, N.A. (1982*a*). The effect of epidermal growth factor (EGF) on cell proliferation of the gastrointestinal mucosa in rodents. *Virchows Arch.* (*Cell Pathol.*), **40,** 63.

AL-NAFUSSI, A.I. & WRIGHT, N.A. (1982*b*). Circadian rhythm in the rate of cellular proliferation and in the size of the functional compartment of mouse jejunal epithelium. *Virchows Arch.* (*Cell Pathol.*), **40,** 71.

BOWER, J.M., CAMBLE, R., GREGORY, H., GERRING, E.L. & WILLSHIRE, I.R. (1975). The inhibition of gastric acid secretion by epidermal growth factor. *Experientia,* **31,** 825.

CARPENTER, G. & COHEN, S. (1979). Epidermal growth factor. *Ann. Rev. Biochem.,* **48,** 193.

CHABOT, J.G., PAYET, N. & HUGON, J.S. (1983). Effects of epidermal growth factor (EGF) on adult mouse small intestine *in vivo* and in organ culture. *Comp. Biochem. Physiol.,* **74A,** 247.

COHEN, S. (1962). Isolation of a mouse submaxillary gland protein accelerating incisor eruption and eyelid opening in the new-born animal. *J. Biol. Chem.,* **237,** 1555.

COHEN, S. & CARPENTER, S. (1975). Human epidermal growth factor: Isolation and chemical and biological properties. *Proc. Natl Acad. Sci.,* **72,** 1317.

COHEN, S. & ELLIOTT, G.A. (1963). The stimulation of epidermal keratinization by a protein isolated from the submaxillary gland of the mouse. *J. Invest. Derm.,* **40,** 1.

COHEN, S. & TAYLOR, J.M. (1974). Part 1. Epidermal growth factor: Chemical and biological characterization. *Recent Progress Hormone Res.,* **30,** 533.

ELDER, J.B., WILLIAMS, G., LACEY, E. & GREGORY, H. (1978). Cellular localisation of human urogastrone/epidermal growth factor. *Nature,* **271,** 466.

FORGUE-LAFITTE, M.G., LABURTHE, M., CHAMBLIER, M.C., MOODY, A.J. & ROSSELIN, G. (1980). Demonstration of specific receptors for EGF urogastrone in isolated rat epithelial cells. *FEBS Lett.,* **114,** 243.

GOSPODAROWICZ, D., GREENBURG, G., BIALECKI, H. & ZETTER, B.R. (1978). Factors involved in the modulation of cell proliferation *in vivo* and *in vitro*: The role of fibroblast and epidermal growth factors in the proliferative response of mammalian cells. *In vitro,* **14,** 85.

GREGORY, H. (1975). Isolation and structure of urogastrone and its relationship to epidermal growth factor. *Nature,* **257,** 325.

HIRATA, Y. & ORTH, D.N. (1979). Epidermal growth factor (urogastrone) in human tissues. *J. Clin. Endocrinol. Metab.,* **48,** 667.

KOFFMAN, C.G., ELDER, J.B., GANGULI, P.C., GREGORY, H. & GEARY, K.G. (1982). Effect of urogastrone on gastric secretion and serum gastrin concentration in patients with duodenal ulceration. *Gut,* **23,** 951.

KONTUREK, S.J., RADECKI, T., BRZOZOWSKI, T. & 6 others (1981). Gastric cytoprotection by epidermal growth factor. Role of endogenous prostaglandins and DNA synthesis. *Gastroenterology,* **81,** 438.

KRIEGER, D. T., HAUSER, H., LIOTTA, A. & ZELENETZ, A. (1976). Circadian periodicity of epidermal growth factor and its abolition by superior cervial ganglionectomy. *Endocrinology,* **99,** 1589.

MALO, C. & MENARD, D. (1982). Influence of epidermal growth factor on the development of suckling mouse intestinal mucosa. *Gastroenterology,* **83,** 28.

McELWAIN, T.J., HEDLEY, D.W., BURTON, G. & 10 others (1979). Marrow autotransplantation accelerates haematological recovery in patients with malignant melanoma treated with high-dose melphalan. *Br. J. Cancer,* **40,** 72.

MILLAR, J.L., HUDSPITH, B.N., McELWAIN, T.J. & PHELPS, T.A. (1978). Effect of high-dose melphalan on marrow and intestinal epithelium in mice pretreated with cyclophosphamide, *Br. J. Cancer,* **38,** 137.

MOORE, G.P.M., PANARETTO, B.A. & ROBERTSON, D. (1981). Effects of epidermal growth factor on hair growth in the mouse. *J. Endocrinol.,* **88,** 293.

SCHEVING, L.A., YEH, Y.C., TSAI, T.H. & SCHEVING, L.E. (1979). Circadian phase-dependent stimulatory effects of epidermal growth factor on deoxyribonucleic acid synthesis in the tongue, esophagus and stomach of the adult male house. *Endocrinology,* **105,** 1475.

SCHEVING, L.A., YEH, Y.C., TSAI, T.H. & SCHEVING, L.E. (1980). Circadian phase-dependent stimulatory effects of epidermal growth factor on deoxyribonucleic acid synthesis in the duodenum, jejunum, ileum, caecum, colon and rectum of the adult male mouse. *Endocrinology,* **106,** 1498.

WITHERS, H.R. & ELKIND, M.M. (1970). Microcolony survival assay for cells of mouse intestinal mucosa exposed to radiation. *Int. J. Radiat. Biol.,* **17,** 261.